# Two Cases of COVID-19 With Concomitant Unexplained Dental Pain: Coincidence or Related?

**DOI:** 10.7759/cureus.35161

**Published:** 2023-02-18

**Authors:** Agness K Rizk, Marwa Jaffal, Wael Khalil

**Affiliations:** 1 General Dentistry, Lebanese University, Beirut, LBN; 2 Restorative and Esthetic Dentistry, Saint Joseph University, Beirut, LBN; 3 Oral and Maxillofacial Surgery, Lebanese University, Beirut, LBN

**Keywords:** nociceptive pain, nociplastic pain, neuropathic pain, pain, neurological symptoms, covid-19

## Abstract

As the rapidly evolving Covid-19 pandemic spread and became a global concern and the study of the disease’s features became possible, its signs and symptoms were elucidated in many studies around the world. In addition to other fairly typical symptoms including fever, cough, sore throat, and shortness of breath, the Covid-19 pandemic has several neurological symptoms that have been noted and documented, including headaches, muscle, and joint discomfort, loss of taste and smell, as well as generalized body aches. However, there were a few unusual symptoms that were noted, one of which we will focus on in this report, namely, tooth pain. The Covid-19 virus has caused tooth pain and discomfort in two Lebanese patients. The reported pain is not related to any local aggression; therefore, it is likely a neurological consequence of the viral infection by the SARS-Cov-2 virus.

## Introduction

The severe acute respiratory syndrome coronavirus 2 (SARS-Cov-2) is responsible for the Covid-19 disease defined by the World Health Organization in February 2020, mediating a global pandemic and a reason for concern [1]. As Covid-19 symptoms can go from mild to severe leading to patients’ hospitalization and admission into care units, the most common symptoms that lead to early detection of the disease are fever, cough, fatigue, and dyspnea [2]. Regarding additional symptoms, a variety of neurological manifestations and complications have been documented. Seizures, meningitis, encephalitis, and acute cerebrovascular events were listed as uncommon complications [2], while headache, dizziness, taste and smell dysfunctions, and pain (headache, joint and muscle pain, etc.) are reported under common manifestations [3]. Tooth pain has never been reported as an associated symptom of Covid-19. Here we report two cases of toothache in two Lebanese women as an unusual manifestation of early-onset coronavirus-2 infection. The International Association for the Study of Pain (ISAP) defines pain as "an unpleasant sensory and emotional experience associated with actual or potential tissue damage or described in terms of such damage" [4], and it is known to be the most common experience reported during health adversities [5]. Regarding pain classifications, there are no single criteria according to which pain can be classified. For example, classifications can be duration-based, causative-agent based, and mechanism-based [6]. The one that is most relevant to our case study is mechanism-based classification. In this classification three categories of pain could be noted: neuropathic pain, which is defined as “pain caused by a lesion or injury of the somatosensory system” [7], the nociceptive pain, which is “pain that arises from actual or threatened damage to non-neural tissue and is due to the activation of nociceptors” [7], and nociplastic pain, which refers to “pain that arises from altered nociception despite no clear evidence of actual or threatened tissue damage causing the activation of peripheral nociceptors or evidence for disease of lesion of the somatosensory system causing the pain” [8].

## Case presentation

Tooth pain is usually caused by tooth decay, dental abscess, dental infection, trauma or tooth damage, etc. However, Covid-19-related tooth pain is rarely reported. In our clinical observation, we have noticed two cases of tooth pain associated with Covid-19 infection and not directly associated with any local cause such as decay, inflammation, or infection.

A 23-year-old female with no reported medical conditions tested positive for Covid-19 in January 2022. Among other symptoms (headache, cough, and congestion), the patient experienced spontaneous pulsatile pain in her left upper incisor that would diminish 45 minutes after taking 1 g of paracetamol. The patient visited her dentist five days after testing negative to check for tooth decay. The second case involves a 16-year-old female undergoing orthodontic treatment, who experienced pain in her right central incisor on the second day of symptoms after testing positive for Covid-19 (December 2022). She was previously diagnosed with hyperthyroidism for which she was taking Tapazole 5mg. A week after testing negative, the patient visited the dentist to check for the cause of the pain: no cavities nor periapical lesions were detected. 

**Figure 1:**
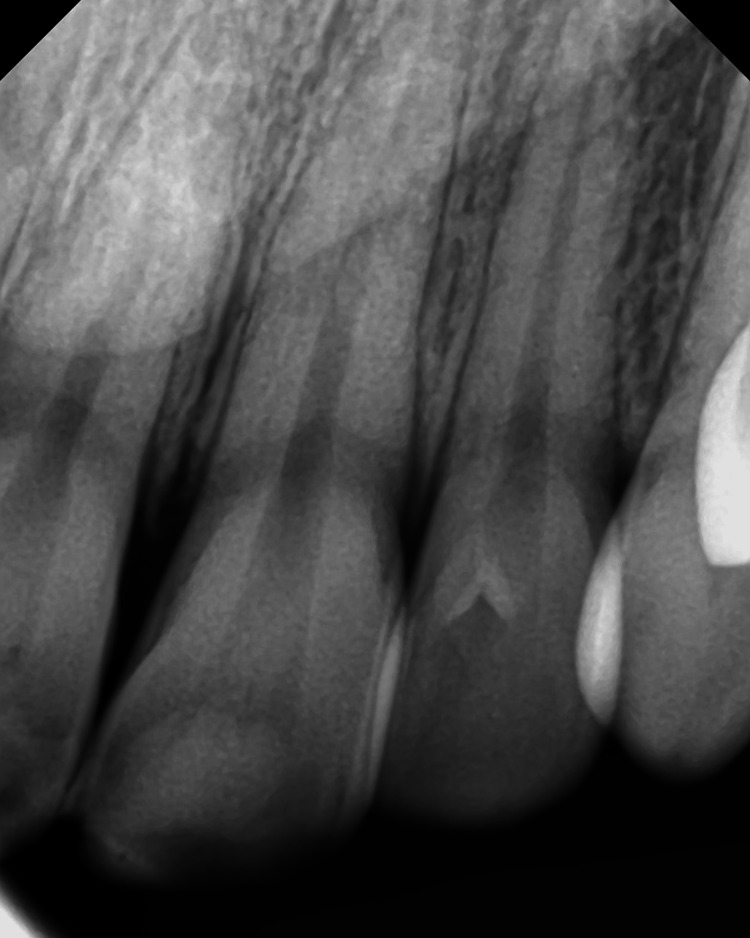
Periapical radiograph of Case #1 showing left lateral incisor with no cavitation. Periapical radiograph taken five days after testing negative for Covid-19.

**Figure 2:**
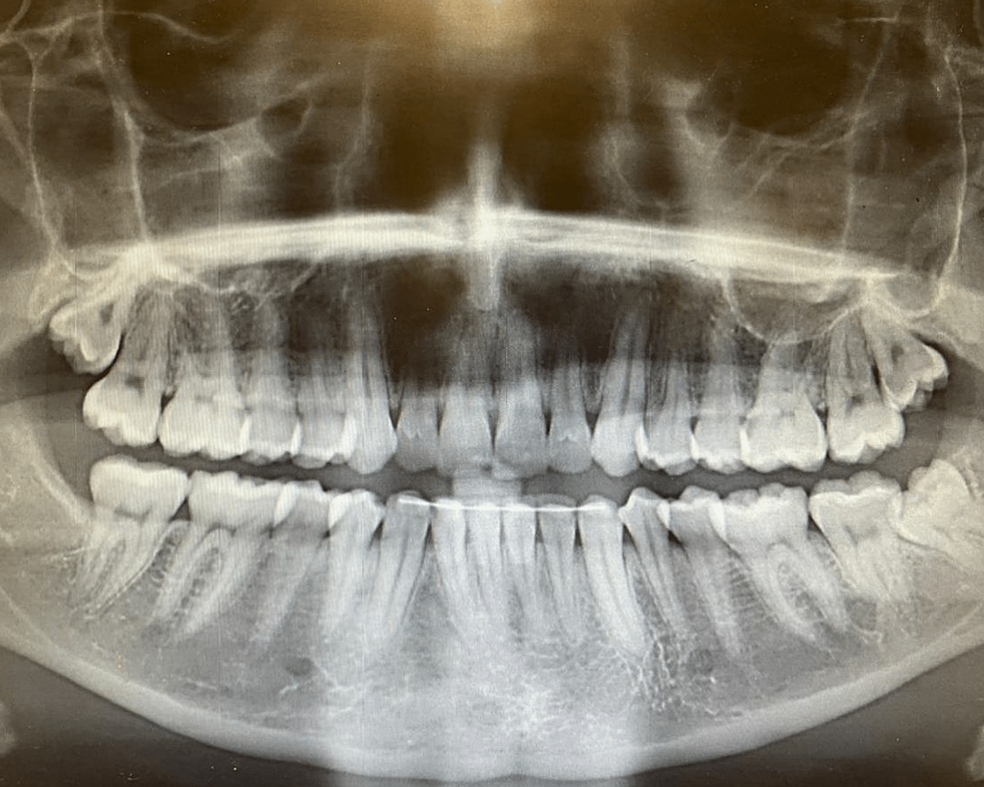
Panoramic radiograph of Case #1 showing left lateral incisor with no cavitations or apical lesions. Panoramic radiograph taken five months after testing negative for Covid-19

**Figure 3:**
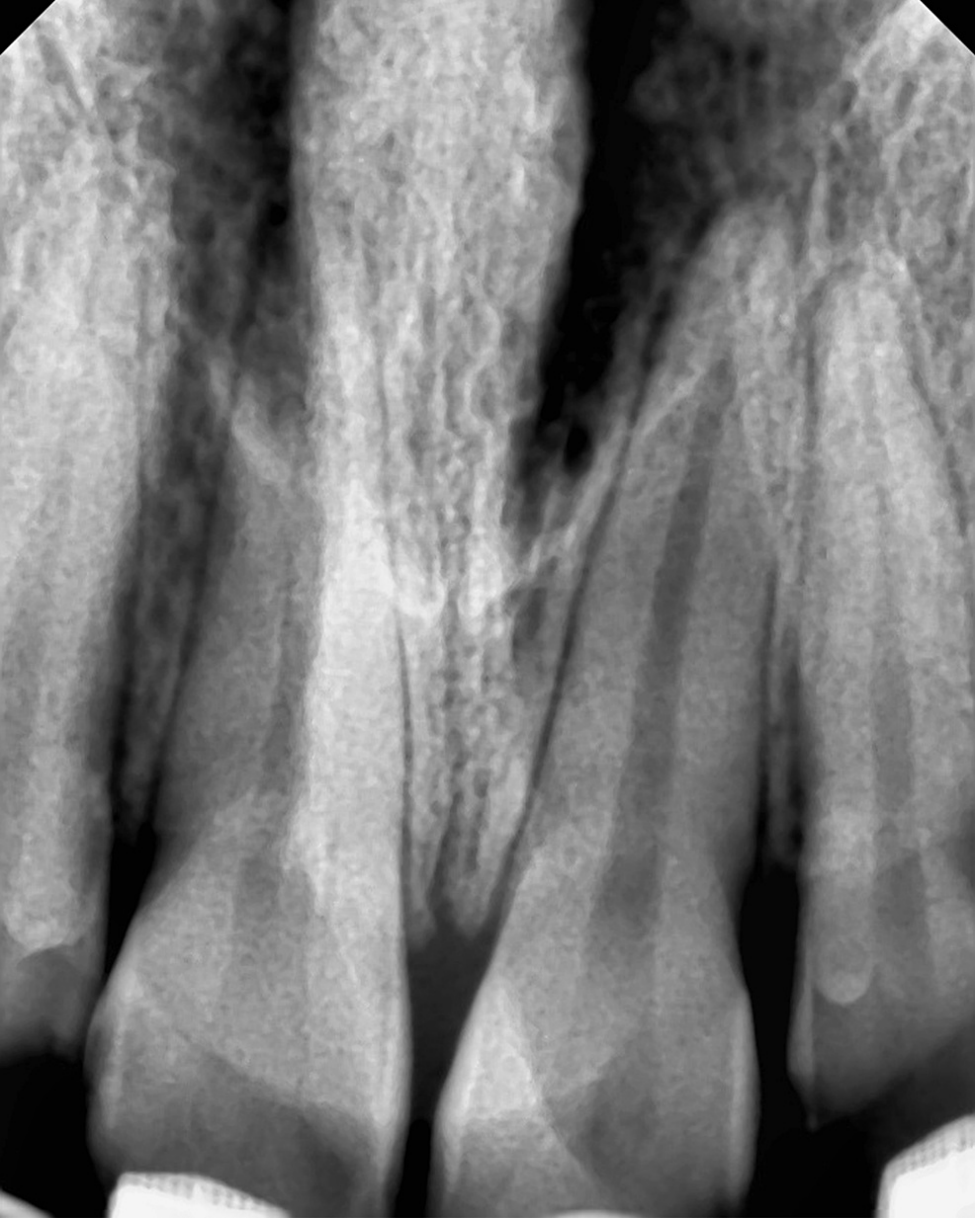
Periapical radiograph of Case #2 showing right central incisor with no cavitations or apical lesions. Periapical radiograph taken seven days after testing negative for Covid-19.

The mentioned cases are hereby presented and summarized in the following table (Table 1) along with a radiographic presentation of Case 1 (Figures 1-2) and Case 2 (Figure 3):

**Table 1:** Summary of the cases included in the study *NPRS stands for numerical rating scale which represents a subjective and unidimensional measurement scale that is represented by a horizontal line that ranges from “0” equivalent to “No Pain” to “10” equivalent to “Worst Pain Ever” [9].

Case number		Age	Gender	Medical history		Tooth in question	Tooth description		Variations	Trigger			Quality	Pain intensity			Response to painkillers		Duration of pain
#1		23	Female	No reported medical conditions		Left upper Lateral incisor	No previous treatment done, no caries were detected.		No variations	No physical trigger, spontaneous Pain			Pulsatile pain	6 out of 10 according to the NPRS*			The pain diminished 40-45 minutes after taking 1g of minor analgesics (Panadol) and the patient remained pain-free for two hours		Pain appeared on the second day of symptoms and remained for three to four days.
#2		16	Female	Diagnosed hyperthyroidism medicated with Tapazole 5mg 1/2 pill/ day.		Right central incisor	The orthodontic bracket was placed on the affected tooth, no caries were detected		No variations.	No physical trigger, spontaneous pain			Pressure pain and electrical shock feeling	6 out of 10 according to the NPRS*			The pain diminished 30-35 minutes after taking 500mg of minor analgesics (Panadol) and the patient remained pain-free for two to three hours.		Pain appeared on the second day of symptoms and remained for four to five days.

## Discussion

As previously mentioned, tooth pain can have multiple causes. One important cause is dentin exposure due to structural compromise of the tooth that comes in the forms of decay, tooth trauma, and tooth wear (erosion, abfraction, etc.). Additionally, infection is one of the main causes behind tooth pain and can be manifested in forms of periodontitis (rarely painful), apical periodontitis that develops into a dental abscess, and pericoronitis that is mostly affiliated with erupting teeth or partially erupted teeth or third molar removal [10]. In the aforementioned cases, the clinical presentation reveals no tooth structure exposure, swelling, or any type of local direct cause for tooth pain; radiological examination also reported no findings. Accordingly, we suggest that a possible explanation of the reported pain is to classify it under neuropathic pain. As defined by the NeuPSIG, neuropathic pain originates from a dysfunction or lesion in the somatosensory system. The causative lesion mentioned in the definition can occur in the peripheral or central nervous system. Similar to the suspected pathogenesis of anosmia in Covid-19 patients that implies the virus damage neurons implicated in olfactory mechanisms [11], tooth pain can occur due to direct damage caused by the virus on the central nervous system, leading to the feeling of tooth discomfort in a particular non-previously damaged tooth. More specifically in the above-reported cases, and since both aching teeth are in the anterior upper arch (left lateral incisor in Case 1 and right central incisor in Case 2), an explanation for the reported pain is a dysfunction in the superior alveolar division of the maxillary nerve V2 (a sensory branch of the fifth cranial nerve: the trigeminal nerve V) that innerves those two teeth [12]. One of the explanations for this pain is that it might be caused by the same neurological dysfunction causing the other common symptoms in Covid-19 patients (like olfactory and gustatory dysfunction). In addition, what further emphasizes our hypothesis is that the pain experienced by both patients used to get relief approximately 30-45 minutes after taking a minor analgesic (paracetamol) and persisted only for four to five days of the period of infection. None of the patients reported any similar pain after their recovery 14 days or more after their first affirmative PCR test demonstrating their infection with Covid-19. All of the findings were indications of neuropathic pain caused by a viral infection, affecting the central nervous system and only indirectly affecting the tooth structures or related nerves.

## Conclusions

In conclusion, tooth pain might be listed as an early symptom of Covid-19 infection and could lead to the diagnosis of Covid-19, based on clinical signs and as a part of neurological disorder, notably in absence of local affections of tooth structures and surrounding tissues (periodontium, sinuses, etc.). These previously mentioned cases may pose the hypothesis of Covid-19 causing neuropathic pain manifested by toothache. In addition, even though this case report presented only two clinical cases with lack of evidence, it might show the dental impact of Covid-19 infection and guide the dental practitioner to spare the patient from having unnecessary dental treatments. Therefore, further investigations should be done on a larger group of patients who experienced similar dental symptoms during their Covid-19 infection.
